# Characteristics and survival outcomes in pediatric patients with spinal chordomas: insights from the National Cancer Database and review of the literature

**DOI:** 10.1007/s11060-024-04921-x

**Published:** 2025-01-02

**Authors:** Victor Gabriel El-Hajj, Sruthi Ranganathan, Rami Rajjoub, Abdul Karim Ghaith, Nicholas Theodore, Adrian Elmi-Terander, Daniel Lubelski

**Affiliations:** 1 Department of Clinical Neuroscience, Karolinska Institutet, Stockholm, Sweden; 2 Department of Medicine, University of Cambridge, Cambridge, UK; 3 Mayo Clinic Neuro-Informatics Laboratory, Mayo Clinic, Rochester, MN USA; 4 Departments of Neurosurgery, Johns Hopkins University School of Medicine, Baltimore, MD USA; 5 Capio Spine Center Stockholm, Löwenströmska Hospital, (Box 2074), Upplands-Väsby, 194 02 Sweden

**Keywords:** Spinal chordoma, Pediatric, Clinical outcomes, National cancer database

## Abstract

**Purpose:**

Spinal chordomas are aggressive tumors that rarely occur in the pediatric population. Demographics and post-treatment outcomes in this select group of patients is poorly studied. We hence aimed to analyze the clinical characteristics, demographics, and survival outcomes of pediatric patients with spinal chordomas, in contrast to the adult population. To address this, the literature was reviewed to evaluate the coverage on spinal chordomas of the pediatric population, and the National Cancer Database (NCDB) was analyzed to provide insights into the US experience over the past two decades.

**Methods:**

A search of the literature was performed leveraging the MEDLINE and Web of Science electronic databases from inception until March 2024, using the keywords “spinal,” “chordoma,” and “pediatric”. Additionally, the NCDB was queried for pediatric patients (≤ 21 years) with chordoma treated between 2004 and 2017. Baseline characteristics, tumor specifics, treatment details, and survival outcomes were collected and analyzed.

**Results:**

From the literature, 45 pediatric chordoma patients were identified, with a median age of 7 years. Most chordomas were in the cervical spine (40%), and 93% of the patients received surgical treatment. Gross total resection was achieved in 59% of cases, and 49% received adjuvant radiotherapy. Recurrence, metastasis, and mortality rates were 7%, 18%, and 24%, respectively at a median follow-up of 12 months. In the NCDB cohort, 53 pediatric patients (≤ 21 years) and 980 adults (> 21 years) were compared. Despite having smaller tumors in size, pediatric patients presented with more advanced tumors with a higher proportion of stage 4 tumors. They had more mobile spine chordomas (83% vs. 51%) and traveled further for treatment (57 vs. 27 miles). Pediatric patients also received higher radiation doses (5420 vs. 5049 cGy). Surgical resection and adjuvant radiotherapy were common treatments in both groups. After matching, outcomes, including survival rates and early mortality, were similar between age groups. Kaplan-Meier analysis showed no difference in overall survival probabilities between the age groups both prior to and after matching.

**Conclusion:**

While pediatric patients with spinal chordomas present with more advanced stage tumors, they demonstrate similar overall survival outcomes when compared to adults. The current literature is mainly composed of single cases and other reports of low evidence levels.

**Supplementary Information:**

The online version contains supplementary material available at 10.1007/s11060-024-04921-x.

## Introduction

Spinal chordomas have an incidence of 0.18–0.84 per million persons per year [1]. These tumors originate from remnants of the primitive notochord that develop along the neuraxis [2], and can be further categorized as sacral chordomas (29–45%) and chordomas of the mobile spine (15–33%) [1]. These tumors are difficult to manage and typically require surgical therapy with maximal safe resection, often combined together with adjuvant radiotherapy. Despite optimal management, spinal chordomas have a high rate of recurrence and are associated with relatively high mortality rates [3–5]. While the majority of patients diagnosed with chordomas are adults between 40 and 60 years of age, occurrences in the pediatric population are not unprecedented [6]. In fact, it is estimated that about 5% of chordomas occur in the first two decades of life [7].

Previous studies found variations in clinical features and anatomical location between adult and pediatric chordomas [8]. Chordomas in younger patients often present with more aggressive histological features, which may result in poorer outcomes [9–11]. Additionally, chordomas of the spine are less commonly represented in pediatric cohorts as compared to adults. A comprehensive study including both pediatric and adult patients reports a significantly lower proportion of chordomas in the sacral region in pediatric, as compared to adult patients [12].

Though a few studies have explored the outcomes associated with chordomas of the skull base in a pediatric population, there are no studies addressing pediatric spinal chordomas, except for case reports [13]. To address this gap, we first conducted a literature review in an attempt to capture the global coverage of this rare disease, then we analyzed the patient characteristics, demographics, and survival outcomes, contrasting pediatric and adult populations with spinal chordomas. For that, data from the National Cancer Database (NCDB), a large US population-based cancer database was utilized.

## Methods

### Data sources

#### Literature review

**Fig. 1:**
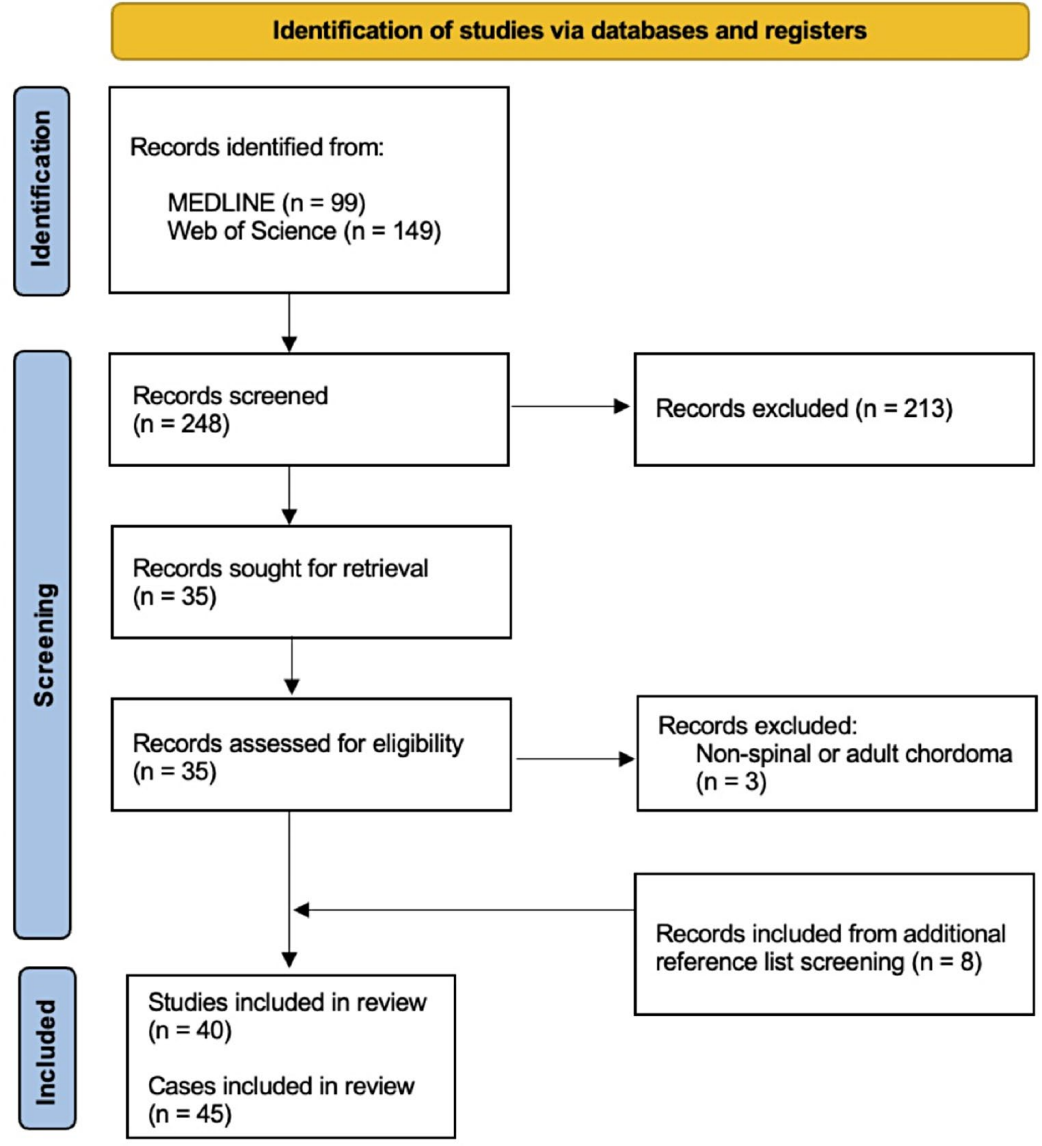
PRISMA flow diagram showing the literature review, search strategy, and study selection process

The PRISMA (Preferred Reporting Items for Systematic Reviews and Meta-Analyses) flow diagram details our study selection process. Initially, a systematic search of the literature was performed on MEDLINE and Web of Science from inception to March 2024, using the keywords “spinal,” “chordoma,” and “pediatric” in all logical permutations (Supplementary file 1). This search identified 248 records. Two authors independently screened these records for eligibility (V.G.E. and S.R.). Conflicts were resolved by discussion followed by unanimous decision. Exclusion criteria included non-English studies, studies on adult patients, and those with chordomas localized outside the spine (including both mobile and sacral spine). Studies were also excluded if they were of an unsuitable publication type (narrative reviews, conference abstracts, book chapters) and/or if individual patient data could not be extracted. All other studies were eligible for inclusion, without any restrictions with respect to the outcome studied. Records were initially screened based on their titles and abstracts, with studies meeting any of the exclusion criteria excluded at this stage. In the second step, the full texts of the remaining records were reviewed to determine their eligibility for inclusion in the review. Reference lists of the eligible articles were also screened to identify articles missed by the initial search. A total of 41 studies were included in the final review (Fig. 1, Supplementary file 2). Data on age, sex, tumor location, treatment used including: surgery, chemotherapy, and radiotherapy, extent of tumor resection, follow-up time, and outcomes including distant metastases, local recurrence, and mortality were extracted from the manuscript included in the review, as available.

#### National Cancer Database (NCDB) analysis

The NCDB is one of the largest cancer registries in the United States and contains almost 34 million cases from over 1500 hospitals. The data is collected from selected health registries accredited by the American College of Surgeons’ Commission on Cancer (https://www.facs.org/quality%20programs/cancer/coc). The NCDB was queried for pediatric patients with chordoma treated between 2004 and 2017. Patients were identified using the International Classification of Diseases for Oncology, 3rd Edition (ICD-O-3). The following codes were used: 9370, 9371, and 9372. All chordomas were considered for the analysis regardless of subtype. Patient baseline characteristics, tumor characteristics, treatment characteristics, and patient outcomes were collected from the NCDB database. Patients with incomplete data were excluded. As per prior evidence [14], a cut-off age of 21 years was selected to define pediatric cases. As such, patients under 21 years of age were classified as the pediatric group, while those over 21 years were designated as the adult control group. Baseline characteristics included age, sex, race, ethnicity, Charlson-Deyo comorbidity index, income quartile, insurance status, and distance to facility. Regarding tumor characteristics, histological subtype, tumor size (cm) on diagnosis, and metastatic status were recorded. Treatment specific data included extent of tumor resection, radiotherapy modality, days to start and duration of radiotherapy, number of fractions, and radiation doses (cGy). Overall survival constituted the primary outcome of the analysis, while length of hospital stay, and 30-day readmission rates were secondary.

### Statistical analysis

Data from the literature was pooled to obtain percentages for categorical variables and ranges for continuous variables. The Shapiro-Wilk test was used to evaluate the normality of the NCDB data. As all continuous data significantly deviated from a normal distribution pattern (Shapiro-Wilk test p value < 0.05), it is presented as a median with interquartile range (IQR) and categorical data as numbers (proportion). The Mann-Whitney U, Chi squared, or Fisher’s exact test were used for between-group comparisons in the NCDB cohort, as appropriate. Propensity score was employed to create balanced comparison groups based on the following covariates: sex, race, ethnicity, extent of tumor resection, tumor size, Charlson-Deyo comorbidity index score, histological subtype, TNM stage, and adjuvant therapy including chemotherapy. The `MatchIt` package in R was utilized to perform a 3:1, leveraging the “optimal” method. After matching, standardized mean difference comparison and Love plots were performed to ensure a balanced distribution of the covariates. An adjusted Kaplan-Meier survival analysis was conducted on the post-matching cohorts to determine the overall survival over follow-up time. The Log-Rank test was performed to assess the significance of the survival analysis. Statistical significance was set at *p* < 0.05 All analyses were conducted using the statistical software program R version 4.0.5 (The R foundation, Austria).

## Results

### Analysis of the available literature

A total of 41 studies on 45 patients were included from our comprehensive search of pediatric chordoma published in existing literature [15–55]. Overall, 20/44 (45%) patients with reported sex were males and the median age was 7 (IQR: 3–10) (Table 1). Most chordomas were in the cervical spine (18/44; 40%), followed by the lumbar (10/44; 23%), sacral (10/44; 23%), and thoracic spine (6/44; 14%). The location was unknown in one patient. The majority of patients received surgical treatment (42/45; 93%). The extent of tumor resection differed across the reported patients, where 20/34 (59%) achieved a gross total resection of the tumor, 12/34 (35.3%) achieved a subtotal resection, and 2/34 (6%) only had a biopsy done. The use of adjuvant radiotherapy was specified in 22 of the patients (49%), while the use of chemotherapy was only reported in 3 patients (7%). Follow-ups were available for 32 of the patients (71%) and ranged from 0.3 months (due to mortality) to 204 months (= 17 years) with a median follow-up time of 12 months (IQR: 8–48).

As for outcomes, local recurrences occurred in 3 (7%), distant metastases in 8 patients (18%), and mortality in 11 patients (24%) at a median follow-up of 12 months.

**Table 1:** Summary of key factors extracted from 45 pediatric patients with spinal chordomas in literature

	Overall (*N* = 45)
**Male sex**	20 (45%)
Missing	1
**Median age in years (IQR)**	8 (3–10)
**Location**	
Cervical	18 (40%)
Thoracic	6 (14%)
Lumbar	10 (23%)
Sacral	10 (23%)
Missing	1
**Surgical treatment**	42 (93%)
**Extent of Resection**	
Biopsy	2 (6%)
Gross total resection	20 (59%)
Subtotal resection	12 (35%)
Missing	11
**Adjuvant radiotherapy**	22 (49%)
**Chemotherapy**	3 (7%)
**Median follow-up in months (IQR)**	12 (8–48)
Missing	12
**Distant metastasis**	8 (18%)
**Local recurrence**	3 (7%)
**Mortality**	11 (24%)

### Analysis of the nationwide cohort from the NCDB

#### Baseline characteristics

The selected, pre-matching cohort included a total of 1033 patients with chordomas of the spine, consisting of 53 pediatric patients (≤ 21 years) and 980 adults (> 21 years). The median age among the pediatric cohort was 17 (12–20), while the median age among the adult cohort was 53 (44–60) (*p* < 0.001; Table 2).

**Table 2:** Demographic and clinical characteristics of adult and pediatric chordoma patients from NCDB

	Adult cohort, *N* = 980	Pediatric cohort, *N* = 53	*p*-value
**Age**	53 (44, 60)	17 (12, 20)	< 0.001
**Male sex**	607 (62%)	19 (36%)	< 0.001
**Race**			0.013
White	875 (91%)	43 (84%)	
Black	48 (5.0%)	1 (2.0%)	
Asian	23 (2.4%)	6 (12%)	
Other	13 (1.3%)	1 (2.0%)	
**Hispanic ethnicity**	64 (6.9%)	5 (10%)	0.38
**Area**			0.80
Metro	783 (88%)	43 (88%)	
Urban	93 (10%)	6 (12%)	
Rural	11 (1.2%)	0 (0%)	
Missing	93	4	
**Distance to treating center (miles)**	31 (12, 103)	57 (14, 284)	0.088
**Charlson comorbidity grade**			0.10
0	829 (85%)	51 (96%)	
1	116 (12%)	1 (1.9%)	
2	30 (3.1%)	1 (1.9%)	
3	5 (0.5%)	0 (0%)	
**Primary site**			< 0.001
Sacrum	480 (49%)	9 (17%)	
Mobile spine	500 (51%)	44 (83%)	
**TMN stage**			0.022
1	462 (87%)	18 (69%)	
2	31 (5.8%)	2 (7.7%)	
3	3 (0.6%)	0 (0%)	
4	35 (6.6%)	6 (23%)	
Missing	449	27	
**Largest tumor diameter (cm)**	53 (33, 85)	30 (23, 55)	0.001
**Treatment**			0.58
Surgery and radiotherapy	748 (76%)	44 (83%)	
Surgery alone	144 (15%)	5 (9.4%)	
Radiotherapy alone	88 (9.0%)	4 (7.5%)	
**EOR (in surgically treated cases)**			0.10
GTR	421 (47%)	16 (33%)	
STR	126 (14%)	7 (14%)	
Unspecified	345 (39%)	26 (53%)	
**Chemotherapy**	27 (2.8%)	4 (7.5%)	0.069
**Days from diagnosis to surgery**	30 (0, 65)	15 (0, 95)	0.48
**Days from diagnosis to radiation**	98 (61, 160)	76 (50, 140)	0.32
**Total radiation doses (cGy)**	5,049 (3,908, 7,000)	5,420 (4,919, 7,395)	**0.024**

There was a significantly lower proportion of males in the pediatric population compared to the adult population (36% vs. 62%; *p* < 0.001). Also, there were significantly fewer White and more Asian patients among the pediatric population as compared to the adult cohort (*p* = 0.013), but no difference in terms of ethnicity (*p* = 0.38). There was no difference in the area of residence amongst the groups, with most of the patients (88%) being in metro areas. Nonetheless, pediatric patients travelled further distances to receive care (57 vs. 27 miles; *p* = 0.045). Although there was a trend towards lower comorbidity levels on the Charlson comorbidity index score in pediatric patients, the difference did not reach statistical significance (*p* = 0.10).

Chordomas of the mobile spine were significantly more common among pediatric patients (83% vs. 51%), whereas chordomas of the sacrum were more common among adults (49% vs. 17%; *p* < 0.001). In terms of the TMN grading, tumors were significantly more advanced in pediatric patients, who exhibited significantly higher proportions of stage 4 tumors (23% vs. 6.6%). Despite that, the largest tumor diameter was significantly smaller in pediatric as compared to adult patients (30 vs. 53 cm; *p* = 0.001).

Surgery with adjuvant radiotherapy was offered in the majority of cases regardless of the age group (83% vs. 76%; *p* = 0.58). Extent of resection was unspecified in around 45% of cases. However, the distribution of reported extent of resections did not differ among groups, with most receiving gross total resection, followed by subtotal resection (*p* = 0.10). Pediatric patients received higher total radiation doses as compared to the adult population (5,420 vs. 5,049 cGy; *p* = 0.024). There was no difference in the time from diagnosis to surgery or diagnosis to radiation treatment, among the groups (*p* ≥ 0.05). Adjuvant systemic chemotherapy was offered in 7.5% of pediatric patients and 2.8% of adults with spinal chordomas (*p* = 0.069).

#### Clinical outcomes

**Fig. 2:**
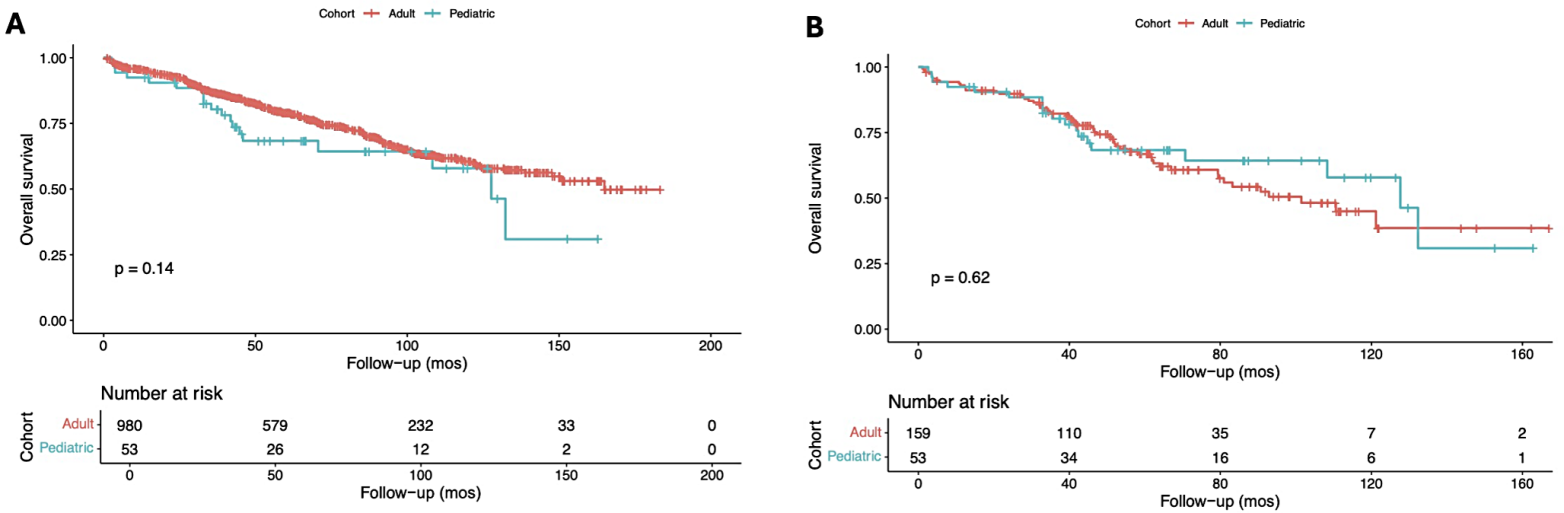
Kaplan-Meier curves comparing mortality in adult and pediatric patients with chordomas of the spine, prior to (**A**), and after propensity score matching (**B**)

After matching, the distribution of baseline patient, tumor, and treatment characteristics was balanced among the groups (*p* ≥ 0.05; **Supplementary file 3**). The post-matching analysis revealed no differences in terms of outcomes based on the age groups (Table 3). There were no differences in the 30-day hospital readmission (5.8% vs. 0%; *p* = 0.12), 30-day mortality (0.8% vs. 2.4%; *p* = 0.42), 90-day mortality (4.7% vs. 2.4%; *p* > 0.99), and length of hospital stay (median: 5 (IQR: 2–9) vs. median: 3 (IQR: 2–6.8); *p* = 0.079). Similarly, Kaplan-Meier analysis (Fig. 2A and B) showed no difference in overall survival probabilities based on the age group both prior to and after propensity score matching (*p* = 0.14; *p* = 0.62, respectively).

**Table 3:** Comparative outcomes in adult and pediatric patients before and after optimal matching

Characteristics	Pre-matching			Post-matching		
	**Adult cohort**, *N* = 980	**Pediatric cohort**, *N* = 53	*p-value*	**Adult cohort**, *N* = 159	**Pediatric cohort**, *N* = 53	*p-value*
30-day unplanned hospital readmission	41 (4.3%)	0 (0%)	0.27	9 (5.8%)	0 (0%)	0.12
30-day mortality	3 (0.4%)	1 (2.4%)	0.19	1 (0.8%)	1 (2.4%)	0.42
90-day mortality	15 (2.0%)	1 (2.4%)	0.57	6 (4.7%)	1 (2.4%)	> 0.99
Length of hospital stays (days)	5.0 (2.5, 10.0)	3.0 (2.0, 6.8)	0.014	5.0 (2.0, 9.0)	3.0 (2.0, 6.8)	0.079

## Discussion

### NCDB database

Review of the NCDB database revealed several key findings related to the management and outcomes of pediatric spinal chordomas. First, despite pediatric patients having smaller sized tumors, these tumors were found to be more advanced at the time of diagnosis, with a significantly higher proportion of stage 4 tumors compared to adults. Previous literature on the topic has indicated that chordomas in younger individuals typically exhibit distinct tumor biology and growth patterns compared to those in older adults [12]. On this note, recent studies on skull base chordomas have suggested that pediatric patients tend to harbor more aggressive and advanced tumors at the time of diagnosis, echoing with the findings from the current study [56, 57].

Some studies have suggested that the more aggressive nature of chordomas in this population may be due to the underlying histopathological differences when comparing to tumors of the adult population. These differences include poorer differentiation and increased cellularity [8, 58].

In addition, this pattern is reminiscent of other bone tumors, such as osteosarcomas [59], where more aggressive behavior are often observed in younger patients compared to adults.

Despite more advanced tumors among pediatric patients, our analysis also revealed that treatment modalities, including the extent of surgical resection and use of adjuvant radiotherapy, were comparable regardless of the age group. Nonetheless, pediatric patients received significantly higher radiation doses, potentially indicating an effort to achieve better local control given the aggressive nature of their disease [14].

Finally, after propensity score matching, there were no significant differences in short-term outcomes, such as hospital readmission and early mortality, nor in long-term survival outcomes between pediatric and adult cohorts. These findings align with previous literature of similar malignancies suggesting that pediatric patients can achieve outcomes comparable to adults despite presenting with more advanced disease [12, 13]. In conclusion, despite notable differences in baseline characteristics between pediatric and adult spinal chordomas—whereby pediatric patients were more likely to present with advanced tumors—no significant differences in post-treatment outcomes were observed. Given the small sample size, it is difficult to determine if tumor location or radiation doses act as confounding factors for this observation.

### Review of the literature

Spinal chordomas are exceedingly rare tumors when it comes to the pediatric population. The literature review revealed a marked paucity of described reports. The earliest among them, dating back to the 1950s, focused on the diagnostic challenges and variable presentations of chordomas in children. One report highlighted a case of chordoma of the sacrococcygeal region in a girl of 5 years treated in 1959, which had initially produced extensive ulceration of the overlying skin in the sacral region. Several other reports of sacrococcygeal chordomas in the pediatric population followed [45, 46], including one by Richards et al. in 1973 which referred to four cardinal radiological features of these tumors: (1) expansion of the sacrum in its lateral and anteroposterior diameters, (2) loculated areas of adjacent bone thinning, (3) trabeculation of undestroyed bone and (4) areas of tumoral calcification.

More recent reports addressing pediatric chordomas of the sacrococcygeal spine emphasize the importance of considering chordomas in the differential diagnoses for pediatric patients with spinal lesions [42]. In the upper thoracic spine, chordomas may be considered in the differential for a posterior mediastinal mass in a child. A case report describing this unusual presentation in a two-year-old male, highlights the diagnostic challenges they pose [53]. Along these lines, a recent literature review on pediatric extraosseous chordomas observed that these tumors have variable presentations, often present with non-specific symptoms, leading to delays in diagnosis [36]. Additionally, in their review the authors highlight the importance of GTR in the management of these tumors. Nonetheless, the authors also address the fact that achieving GTR may not always be possible, hence reflecting on the central role of adjuvant radiotherapy in the treatment of chordomas. This is also the object of the conclusion of another report of two cases of chordomas of the cervical spine [26], where Choi et al. underscore the need for an aggressive surgical approach when dealing with spine chordomas. The authors also noted that despite aggressive surgical management, recurrence is still a significant risk, necessitating adjuvant radiotherapy to improve local control as well as close postoperative monitoring [26].

While not always feasible, the advance of surgical techniques has enabled en-bloc tumor resection utilizing complex grafting techniques, thereby improving survival outcomes. In a case from Killampalli et al., the authors demonstrate the long-term efficacy of vertebrectomy and fibula strut graft reconstruction in a 7-year-old patient with a chordoma of the lumbar spine [35]. Using advanced surgical techniques to achieve complete resection of the tumor, functional recovery and long-term disease control were achieved [35].

While chordomas of the skull base in pediatric patients have been covered in the literature, there have been no complementary comprehensive analyses on their spinal counterparts [13, 14]. The current literature mainly relies on low level evidence and high risk of bias reports, which provide moderate insights into the clinical characteristics, treatments, and outcomes of pediatric spinal chordomas.

Our review of the literature yielded 41 reports on 45 pediatric patients with chordomas of the spine. Overall, a slight overrepresentation (55%) of female patients was reported with a median age of 7 (IQR: 3–10). While females were also overrepresented in the NCDB cohort (64%), the patients were found to be older with a median age reaching 17 (IQR: 12–20). Most chordomas were located in the cervical spine (40%), followed by the lumbar (23%), sacral (23%), and thoracic spine (14%). In comparison, the NCDB cohort revealed a similarly low proportion of pediatric patients with chordomas of the sacrum (17%), a finding that seem to be consistent across previous literature [12]. The majority of patients received surgical treatment (93%), which was also highlighted in the NCDB, where 93.5% of patients received surgery with or without radiotherapy. On this note, the use of adjuvant radiotherapy was notably lower in the literature compared to the current US-based cohort (49% vs. 83%). This discrepancy may be attributed to differences in treatment timelines, as the NCDB data encompassed only the past two decades, while the cases from the literature search included patients treated in earlier eras. Adjuvant chemotherapy, on the other hand, was administered at similar rates in both pooled literature and NCDB cohorts (7% vs. 7.5%). As for the extent of tumor resection, STR was achieved in 35.3% of pediatric patients in the literature while GTR was achieved in 59% of patients. The remainder of the patients (6%) only had a biopsy done. A systematic review on adult spinal chordoma reported similar findings, with rates of GTR reaching 55% despite demographic differences [2].

In terms of outcomes, locoregional recurrences were noted in 7%, metastases in 18%, and mortality in 24% of the patients, on a median follow-up of 12 months (IQR: 8–48). The low rate of local recurrences likely reflects the inadequate follow-up time, while the high metastasis and mortality rates are likely the result of publication bias. This suggests that the results of this review are to be carefully interpreted.

### Limitations

First, this study utilizing the NCDB database is limited by its retrospective nature. While the NCDB focuses on post-treatment survival outcomes, it lacks other valuable metrics such as locoregional recurrence and patient-reported outcome measures, which could hence not be studied. In addition, the literature review conducted is not exhaustive and was biased towards English-written literature due to the restricted number of databases searched and the language restriction imposed on the search. In addition, all relevant studies included were case reports and are deemed to have high levels of bias. This reflects the rarity of the tumor but also limits the level of evidence retained from the conclusions of the review. Moreover, the studies included in the literature review span from the 1940s to 2024, which introduces variability due to advancements in surgical techniques, imaging modalities, and reporting standards over time. This temporal heterogeneity may introduce potential bias and limit the comparability of outcomes reported across studies. On a similar note, both the heterogeneity and the disparities in time and practices hinder the comparability between NCDB and literature review cohorts. Resulting comparisons between the cohorts are hence to be cautiously interpreted. Nonetheless, this is the first collection of reports pediatric spinal chordomas to date, and we also present the first and largest cohort study describing the mortality rates in this patient group, contrasted with adult controls.

## Conclusion

While pediatric patients with spinal chordomas present with more advanced stage tumors, they demonstrate similar overall survival outcomes when compared to adults. Treatment modalities offered, including surgical resection and adjuvant radiotherapy, were similar across age groups, with pediatric patients receiving higher radiation doses. The current literature is mainly composed of single cases and other reports of low evidence levels. Future multicenter studies with larger sample sizes are needed to confirm these findings and improve treatment strategies for pediatric spinal chordomas.

## Electronic supplementary material

Below is the link to the electronic supplementary material.

Supplementary Material 1

Supplementary Material 2

Supplementary Material 3

## Data Availability

No datasets were generated or analysed during the current study.
