# Supplementary material for: Characteristics and survival outcomes in pediatric patients with spinal chordomas: insights from the National Cancer Database and review of the literature
Source: J Neurooncol. 2025 Jan 2;172(2):397–405. doi: 10.1007/s11060-024-04921-x (PMC11937186; doi:10.1007/s11060-024-04921-x)
Supplement: Supplementary file 1 — Supplementary Material 1 [file 11060_2024_4921_MOESM1_ESM.docx]

**PubMed:**

(Child[MeSH Terms] “Infant”[mesh] OR Adolescent[MeSH Terms] OR Pediatrics[MeSH] OR child*[tiab] OR infant*[tiab] OR newborn*[tiab] OR neonat*[tiab] OR adolescen*[tiab] OR toddler*[tiab] OR teen*[tiab] OR boy[tiab] OR boys[tiab] OR girl*[tiab] OR pediatric*[tiab] OR paediatric*[tiab] OR youth*[tiab]) AND (“chordoma”[mesh] OR “chordoma”[all fields] OR “chordomas”[all fields] OR chordoma*) AND (“spin*”[all fields] OR “spinal”[all fields]) AND (“English"[Language])

**Web of Science:**

((ALL=("spinal chordoma") AND LA=(English)) NOT (TASCA==("VETERINARY SCIENCES") OR DT==("EDITORIAL MATERIAL" OR "LETTER" OR "MEETING ABSTRACT"))) OR ((TI=(spinal AND chordoma*) AND LA=(English)) NOT (TASCA==("VETERINARY SCIENCES") OR DT==("EDITORIAL MATERIAL" OR "LETTER" OR "MEETING ABSTRACT")))
