# Supplementary material for: Characteristics and survival outcomes in pediatric patients with spinal chordomas: insights from the National Cancer Database and review of the literature
Source: J Neurooncol. 2025 Jan 2;172(2):397–405. doi: 10.1007/s11060-024-04921-x (PMC11937186; doi:10.1007/s11060-024-04921-x)
Supplement: Supplementary file 2 — Supplementary Material 2 [file 11060_2024_4921_MOESM2_ESM.docx]

**Supplementary Table 2.** Studies included in the literature review

| **Title** | **First Author** | **Year** | **Article type** | **Patient Age** | **Sex** |
| --- | --- | --- | --- | --- | --- |
| A case report of Sustained triumph: 15-year recurrence-free survival following surgical resection of a cervical chordoma in a 15-year-old girl | Haddad | 2023 | case report | 15 | F |
| An 8-year-old boy with neck pain | Hosalkar | 2002 | case report | 8 | M |
| Anatomic relationship of the internal carotid artery to the C1 vertebra: A case report of cervical reconstruction for chordoma and pilot study to assess the risk of screw fixation of the atlas | Currier | 2003 | case report | 13.5 | F |
| Benign and Malignant Chordomas | Congdon | 1952 | case report | 16 | F |
| Cervical chordoma in a patient with tuberous sclerosis presenting with shoulder pain | Storm | 2007 | case report | 16 | F |
| Cervical chordoma in childhood without typical vertebral bony destruction: case report and review of the literature | Zhou | 2010 | case report | 11 | F |
| Cervical spine chordoma | Hart | 2012 | case report | 13 | F |
| Chordoma in the cervical spine managed with en bloc excision | Fujita | 1999 | case report | 16 | M |
| Chordoma presenting as a posterior mediastinal mass in a pediatric patient | Ahrendt | 1992 | case report | 9 | M |
| Chordoma presenting as a posterior mediastinal mass: A choristoma | Clemons | 1973 | case report | 14 | F |
| Chordoma: A case report | Jallo | 1997 | case report | 13 | F |
| Chordoma. 35-year study at Memorial Hospital. Cancer 20: 1841-1850 (1967) | Higinbotham | 1967 | case report | 2.5 | F |
| Complex cervical spine neoplastic disease: reconstruction after surgery by using a vascularized fibular strut graft. Case report | Wright | 1999 | case report | 15 | F |
| Extraosseous spinal chordoma: radiographic appearance | Sebag | 1993 | case report | 6.5 | M |
| Intrathoracic chordoma presenting as a posterior superior mediastinal tumor | Cotler | 1983 | case report | 14 | F |
| L2 chordoma in an 11-year-old girl | Bedi | 2010 | case report | 11 | F |
| Management and outcome of chordomas in the pediatric population: The Hospital for Sick Children experience and review of the literature | Tsitouras | 2016 | case report | 10 | F |
| Management and outcome of chordomas in the pediatric population: The Hospital for Sick Children experience and review of the literature | Tsitouras | 2016 | case report | 10 | F |
| Management and outcome of chordomas in the pediatric population: The Hospital for Sick Children experience and review of the literature | Tsitouras | 2016 | case report | 10 | F |
| Multilevel oblique corpectomies as an effective surgical option to treat cervical chordoma in a young girl | Delfini | 2014 | case report | 14 | F |
| Multiple epidural lumbar chordomas without bone involvement in a 17-year-old female: a case report | Simon | 2011 | case report | 17 | F |
| Notochordal tumour of the cauda equina in a child of 8 years | Ellis | 1935 | case report | 8 | F |
| Pathological case of the month. Spinal chordoma | Norris | 1992 | case report | 13 | M |
| Pediatric cervical chordoma: report of two cases and a review of the current literature | Choi | 2010 | case report | 7 | M |
| Pediatric cervical chordoma: report of two cases and a review of the current literature | Choi | 2010 | case report | 10 | M |
| Pediatric extraosseous sacral chordoma: case report and literature review of embryonic derivation and clinical implications | Hamilton | 2019 | case report | 11 | M |
| Pediatric metastatic sacrococcygeal chordoma treated with surgery | Al-Adra | 2011 | case report | 12 | M |
| Preadolescent presentation of a lumbar chordoma: results of vertebrectomy and fibula strut graft reconstruction at 8 years | Killampalli | 2006 | case report | 7 | F |
| Review of Pediatric Extraosseous Chordomas with a Unique, Illustrative Case | Lee | 2023 | case report | 11 | F |
| Sacral chordoma--a case report | Khambekar | 1997 | case report | 5 | F |
| Sacrococcygeal and vertebral chordomas and their treatment | Rosenqvist | 1959 | case report | 4 | M |
| Sacrococcygeal chordoma in a 9-year-old boy | de Noronha | 1995 | case report | 9 | M |
| Sacrococcygeal chordoma in a neonate with multiple anomalies | Nix | 1978 | case report | 0 | F |
| Sacrococcygeal chordoma in children | Montgomery | 1933 | case report | 3 | F |
| Sacrococcygeal chordoma--two cases with unusual features | Worthy | 1965 | case report | 5 | F |
| Sacrococcygeal Chordomas in children | Richards | 1973 | case report | 1 | M |
| Sacrococcygeal Chordomas in children | Richards | 1973 | case report | 3 | M |
| Spinal chordomas | Sundaresan | 1979 | case report | 2.5 | NA |
| Spinal chordomas in infancy. Report of a case and analysis of the literature | Occhipinti | 1981 | case report | 4 | M |
| Surgical management of sacrococcygeal and vertebral chordoma | Mixter | 1940 | case report | 14 | M |
| Thoracic intramedullary chordoma without bone involvement: a rare clinical entity | Faheem | 2016 | case report | 8 | M |
| Tuberous sclerosis: A case report with aortic aneurysm and unusual rib changes | Dutton | 1975 | case report | 0 | M |
| Unusual case of nasal obstruction | Swords | 2017 | case report | 15 | M |
| Unusual extradural chordoma in an adolescent presenting with lumbar radiculopathy | Chau | 2020 | case report | 17 | M |
| Unusual presentation of posterior mediastinal chordoma in a 2-year-old boy | Huang | 2003 | case report | 2 | M |
