# Supplementary material for: Characteristics and survival outcomes in pediatric patients with spinal chordomas: insights from the National Cancer Database and review of the literature
Source: J Neurooncol. 2025 Jan 2;172(2):397–405. doi: 10.1007/s11060-024-04921-x (PMC11937186; doi:10.1007/s11060-024-04921-x)
Supplement: Supplementary file 3 — Supplementary Material 3 [file 11060_2024_4921_MOESM3_ESM.docx]

**Table 2**

| **Characteristic** | **post-matching** | | |
| --- | --- | --- | --- |
|  | **Adult cohort**, N = 159*^1^* | **Pediatric cohort**, N = 53*^1^* | **p-value***^2^* |
| **AGE** | 54.0 (40.0, 59.0) | 17.0 (12.0, 20.0) | <0.001 |
| **Male sex** | 61 (38%) | 19 (36%) | 0.74 |
| **Race** |  |  | 0.058 |
| White | 139 (91%) | 43 (84%) |  |
| Black | 7 (4.6%) | 1 (2.0%) |  |
| Asian | 4 (2.6%) | 6 (12%) |  |
| Other | 2 (1.3%) | 1 (2.0%) |  |
| (Missing) | 7 | 2 |  |
| **Hispanic ethnicity** | 19 (13%) | 5 (10%) | 0.60 |
| (Missing) | 13 | 4 |  |
| **Area** |  |  | 0.15 |
| Metro | 139 (95%) | 43 (88%) |  |
| Urban | 7 (4.8%) | 6 (12%) |  |
| Rural | 1 (0.7%) | 0 (0%) |  |
| (Missing) | 12 | 4 |  |
| **Distance to treating center (miles)** | 27.2 (11.5, 83.8) | 57.0 (13.5, 283.9) | 0.045 |
| **Charlson comorbidity grade** |  |  | 0.26 |
| 0 | 148 (93%) | 51 (96%) |  |
| 1 | 10 (6.3%) | 1 (1.9%) |  |
| 2 | 1 (0.6%) | 1 (1.9%) |  |
| 3 |  |  |  |
| **Primary site** |  |  | 0.62 |
| Sacrum | 32 (20%) | 9 (17%) |  |
| Mobile spine | 127 (80%) | 44 (83%) |  |
| **TMN stage** |  |  | 0.86 |
| 1 | 59 (74%) | 18 (69%) |  |
| 2 | 7 (8.8%) | 2 (7.7%) |  |
| 3 | 0 (0%) | 0 (0%) |  |
| 4 | 14 (18%) | 6 (23%) |  |
| (Missing) | 79 | 27 |  |
| **Largest tumor diameter (cm)** | 30.0 (25.0, 60.0) | 30.0 (23.0, 55.0) | 0.64 |
| **Treatment** |  |  | 0.95 |
| Surgery and radiotherapy | 128 (81%) | 44 (83%) |  |
| Surgery alone | 18 (11%) | 5 (9.4%) |  |
| Radiotherapy alone | 13 (8.2%) | 4 (7.5%) |  |
| **EOR (in surgically treated cases)** |  |  | 0.83 |
| GTR | 41 (28%) | 16 (33%) |  |
| STR | 22 (15%) | 7 (14%) |  |
| Unspecified | 83 (57%) | 26 (53%) |  |
| **Chemotherapy** | 9 (5.7%) | 4 (7.5%) | 0.74 |
| **Days from diagnosis to surgery** | 13.0 (0.0, 56.0) | 15.0 (0.0, 95.0) | 0.54 |
| **Days from diagnosis to radiation** | 100.0 (56.0, 162.0) | 76.0 (50.0, 140.0) | 0.43 |
| **Total radiation doses (cGy)** | 5,000.0 (2,540.0, 6,459.0) | 5,238.0 (4,250.0, 7,290.0) | 0.16 |
